# Mixed-Generation PAMAM G3-G0 Megamer as a Drug Delivery System for Nimesulide: Antitumor Activity of the Conjugate Against Human Squamous Carcinoma and Glioblastoma Cells

**DOI:** 10.3390/ijms20204998

**Published:** 2019-10-09

**Authors:** Magdalena Zaręba, Przemysław Sareło, Marta Kopaczyńska, Agata Białońska, Łukasz Uram, Małgorzata Walczak, David Aebisher, Stanisław Wołowiec

**Affiliations:** 1 Faculty of Chemistry, Rzeszów University of Technology, 35-939 Rzeszów, Poland; magzar@prz.edu.pl (M.Z.); luram@prz.edu.pl (Ł.U.); mwalczak@prz.edu.pl (M.W.); 2 Department of Biomedical Engineering, Wrocław University of Science and Technology, 50-370 Wrocław, Poland; przemyslaw.sarelo@pwr.edu.pl (P.S.); marta.kopaczynska@pwr.edu.pl (M.K.); 3 Faculty of Chemistry, University of Wrocław, 50-383 Wrocław, Poland; agata.bialonska@gmail.com; 4 Centre for Innovative Research in Medical and Natural Sciences, Faculty of Medicine, University of Rzeszów, 35-310 Rzeszów, Poland; daebisher@ur.edu.pl

**Keywords:** drug delivery system, PAMAM megamer, nimesulide conjugate, anticancer activity

## Abstract

Polyhydroxylated dendrimer was synthesized from poly(amidoamine) (PAMAM) dendrimer generation 3 by addition of glycidol (**G3^gl^**). **G3^gl^** megamer was further modified by binding PAMAM G0 dendrimers by activation of **G3^gl^** with *p*-nitrophenylchloroformate, followed by the addition of excess PAMAM G0 and purification using dialysis. The maximum G0 binding capacity of **G3^gl^** was 12 in the case when G0 was equipped with two covalently attached nimesulide equivalents. Nimesulide (**N**) was converted into N-(*p*-nitrophenyl) carbonate derivative and fully characterized using X-ray crystallography and spectral methods. Nimesulide was then attached to G0 via a urea bond to yield **G0^2N^**. The mixed generation **G3^gl^–G0^2N^** megamer was characterized using ^1^H NMR spectroscopy, and its molecular weight was estimated to be 22.4 kDa. The AFM image of **G3^gl^–G0^2N^** deposited on mica demonstrated aggregation of nimesulide-covered megamer. The height of the deposited megamer was 8.5 nm. The megameric conjugate with nimesulide was tested in vitro on three human cell lines: squamous cell carcinoma (SCC-15) and glioblastoma (U-118 MG) overexpressing cyclooxygenase-2 (COX-2), and normal skin fibroblasts (BJ). The conjugate efficiently penetrated into all cells and was more cytotoxic against SCC-15 than against BJ. Moreover, the conjugate produced a strong and selective antiproliferative effect on both cancer cell lines (IC_50_ < 7.5 µM).

## 1. Introduction

Nonsteroidal anti-inflammatory drugs (NSAID) are used for treatment of pain and inflammation [1]. Some of them, like celecoxib (**C**) or nimesulid (**N**) are selective or preferential cyclooxygenase-2 (COX-2) inhibitors. Over-expression of COX-2 has been detected in a variety of human tumors in breast, prostate, lung, skin and colon, and therefore, **C** and **N** are currently being tested for anticancer activity [2].

Side effects that can occur during long-term oral administration of NSAID include gastrointestinal irritation, ulcers, and impairment of renal blood circulation and glomerulic filtration. These adverse effects can be avoided by blood administration of **C** and **N** attached to a macromolecular carrier. We have followed the enhanced permeation and retardation (EPR) strategy and recently tested **C** and F-Moc-L-Leucine (**L**), the PPAR-γ agonist attached to third generation polyamidoamine dendrimer (PAMAM G3), in vitro for cytotoxicity against the cancer lines glioblastoma (U-118 MG) and squamous cell carcinoma (SCC-15). We found that the biotinylated conjugate bearing both **C** and **L** showed additive cytotoxicity for fibroblasts and both cancer lines in order BJ > U-118 MG > SCC-15, with IC_50_ in the range 0.69, 1.44 and 2.22 µM, respectively [3]. In addition, we also applied biotinylated PAMAM G3 as carrier of **N** [4]. The bioconjugates **G3^N^** bearing 18 or 31 **N** exerted selective toxic effect on SCC-15 cells but not against normal human fibroblasts (BJ) at low range of concentration (1.25–10 µM) with 250 fold stronger action than native nimesulide. Moreover, this conjugates induced apoptotic cell death via COX-2/PGE_2_ independent pathway.

Full generation PAMAM dendrimers are systematically toxic; therefore, surface amine group modification by PEG-ylation [5], hydroxylation with D-glucoheptono-1,4-lactone [6] or hydroxyalkylation with glycidol is often explored to reduce the systemic toxicity of carrier.

On the other hand, amine groups of full generation PAMAM dendrimers are convenient sites for linking drug molecules or combinations of drugs, targeting molecules like biotin, folate and others.

PAMAM dendrimers can be stored in methanol at low temperatures, nonetheless, after several months, spontaneous aggregation of pristine dendrimers is observed [7]. The aggregation can be performed efficiently under chemical control using an appropriate linker, like glutaraldehyde [8,9], cyclooctyne-azide click coupling [10], or a straightforward reaction between variably terminated dendrimers, like G2-COOH and G2-NH_2_ [7,11]. The stoichiometry and aggregation level in these procedures cannot be strictly controlled; therefore, high molecular weight dispersion of megamers is an inevitable feature.

We began our contribution to the field by using regular PAMAM G3 dendrimer as carrier of retinal, biotin and pyridoxal for transdermal delivery [12,13] and later applied them as carriers for anticancer drugs [3]; glucoheptoamidated PAMAM G3 dendrimer was shown to be an effective carrier for doxorubicin [6,14].

Encouraged by the results on the selective and efficient anticancer activity of PAMAM G3^N^ conjugates, we synthesized a new macromolecular carrier for **N**, comprised of hydroxylated PAMAM G3 with covalently bound PAMAM G0 dendrimers substituted with **N**. In this way, we obtained a very well water soluble megameric conjugate and tested it for cytotoxicity against human squamous carcinoma (SCC-15) and glioblastoma cells (U-118 MG), and comparatively against normal human fibroblasts (BJ).

## 2. Results and Discussion

### 2.1. Chemistry

#### 2.1.1. Reaction of PAMAM Dendrimers with Glycidol

2,3-Epoxypropanol (glycidol, **gl**) is an oxirane which undergoes facile reaction with amine groups. The addition leads to **gl** epoxide ring opening and doubles the number of free hydroxyl groups. Therefore, **gl** found application in obtaining water-soluble oligoetherols from 1,3,5-triazine or barbituric acid, which were further used to obtain polyurethane foams with improved thermal resistance [15]. 

**Scheme 1:**
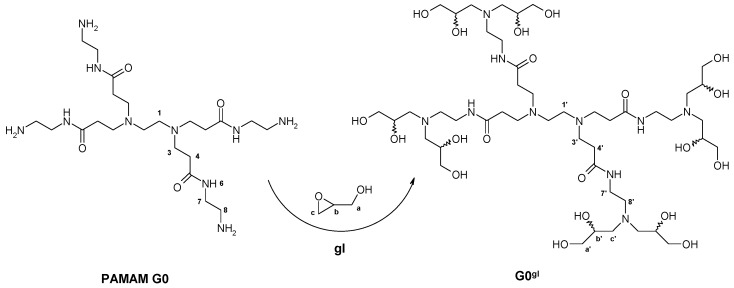
Conversion of polyamidoamine (PAMAM) G0 dendrimer to G0^gl^ by addition of eight glycidol equivalents.

**Figure 1:**
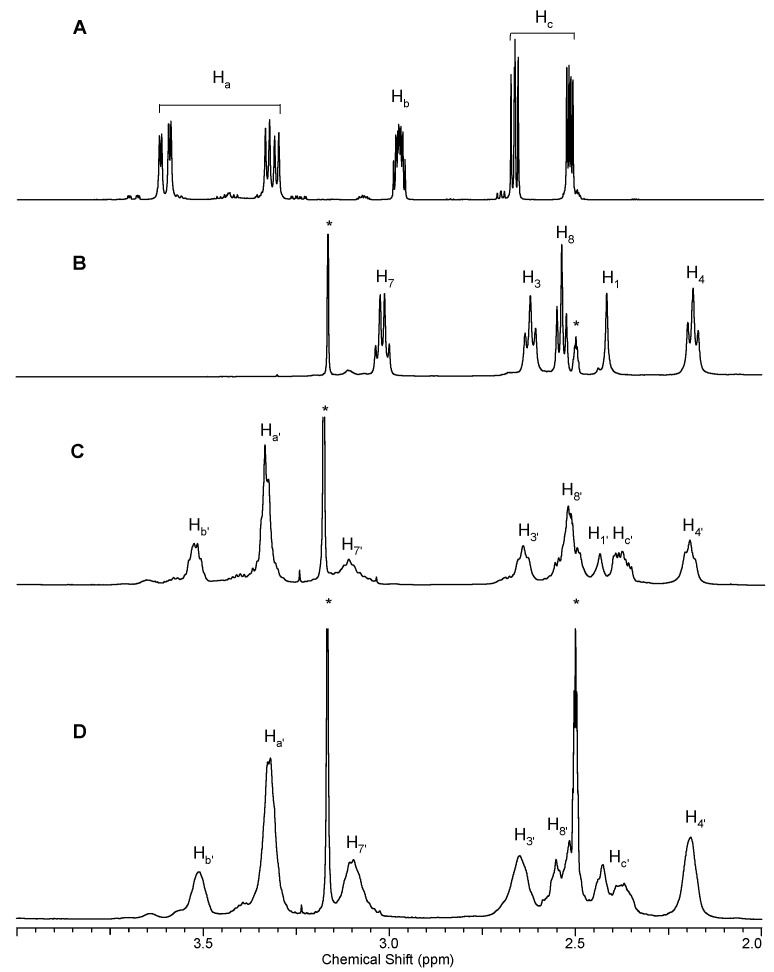
The relevant fragments of ^1^H NMR spectra of: **A**—glycidol, **B**—PAMAM G0, **C**—**G0^gl^**, and **D**—**G3^gl^** in DMSO-d_6_. Resonances are labeled according to atom numbering in Scheme 1, Scheme 2, and Scheme 3. Residual CD_3_SOCD_2_**H** resonance and methanol are labeled with an asterisk. The ^1^H resonances were assigned by COSY experiment.

We have used **gl** to convert PAMAM **G3** dendrimer into a completely hydroxyalkylated derivative, which remained water soluble like starting **G3** and has no free primary amine groups. In order to establish the protocol for this conversion, we present a synthetic path starting from PAMAM **G0**. We found that eight molecules of **gl** are added within two days of reaction with **G0** at room temperature to give the yellow oily product **G0^gl^** (Scheme 1). The progress of reaction was monitored using ^1^H NMR spectroscopy. The spectra of substrates **gl** and **G0** are presented at Figure 1 (traces A and B, respectively). Large magnetic non-equivalence of both a and c methylene groups vicinal to chiral carbon b in **gl** (trace A) is almost cancelled after ring opening and addition of **gl** to **G0**. The largest chemical shift (0.5 ppm downfield) occurs for the H_b_ proton upon conversion from **gl** into 1,2-dihydroxypropyl group in **G0^gl^**. The H_7_ resonance of **G0** resonances is also shifted 0.1 ppm downfield upon addition of two **gl** onto the terminal nitrogen atom of **G0^gl^** (trace C versus B). 

**Scheme 2:**
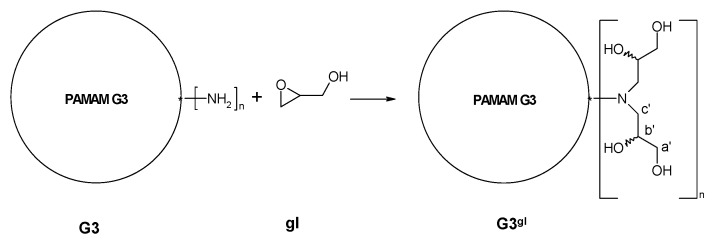
Conversion of PAMAM G3 dendrimer to **G3^gl^** by hydroxyalkylation with glycidol. PAMAM G3 has 32 terminal amine groups (*n* = 32) that each react with two molecules of **gl** to give a total of 64 2,3-dihydroxypropyl substituents with 128 free hydroxyl groups.

The same protocol was used to convert **G3** into **G3^gl^** (Scheme 2). The product was purified by extensive dialysis against water and characterized using ^1^H NMR spectroscopy. The spectrum of **G3^gl^** is shown in Figure 1, trace D. The spectrum of **G3^gl^** is similar to that of **G0^gl^** except for the intensities of resonances from the 2,3-dihydropropyl protons, which correspond to [64H] for b’ and [128H] for a’and c’ in comparison with PAMAM G3 –C**H**_2_-CO- resonance of intensity [120H] at 2.2 ppm (H-4’). The total intensity of carbon-attached ^1^H resonances from **G3^gl^** is [804H], including [484H] from the G3 core and [320H] from peripheral glycidol-derived 2,3-dihydroxypropyl substituents. 

The ^1^H and ^13^C NMR spectra of both **G0^gl^** and **G3^gl^** are quite complicated due to the racemic **gl** substrate used for synthesis. Therefore many stereoisomers are formed at every terminal di-substituted nitrogen, containing an attached chiral **gl** in combinations *RR*, *SS*, *RS*, and *SR* local chirality. When considering only four microstates, two spectra of **gl** substituents can be expected—one from two *meso* forms and a second from chiral *RR* and *SS* microstates. Because more than two spectra are observed, it is obvious that two arms of the outer sphere of dendrimer influence the symmetry of NMR spectra. Namely, 16 microstates must be considered: *RRRR*; *RRRS*; …*RSSS*; *SSSS*, from which six various spectra of relative intensity, 1:1:1:1:2:2, should be observed if no enantiodiscrimination of chiral **gl** addition occurs. Still, the ^13^C NMR spectra show at least four sets of resonances. Additionally, their intensities do not follow the 1:1:1:1:2:2 ratio indicating that a stereochemical discrimination factor for a second **gl** addition influences the populations of microstates. We are currently working on determination of **gl** addition enantioselectivity. Despite the structural complexity of **G3^gl^**, the chemical availability of the terminal hydroxyl group of the 2,3-dihydroxypropyl substituents for further functionalization enables further expansion of the **G3^gl^** dendrimer.

#### 2.1.2. Nimesulide Activation and Attachment to PAMAM G0

*p*-Nitrophenyl chloroformate (NPCF) provides a one-carbon linker to amine groups of PAMAM dendrimers. It has been shown that the copolymer obtained from butylene oxide and ethylene oxide was efficiently activated with NPCF, followed by covalent attachment of PAMAM G1–G3 dendrimers [16]. 

**Scheme 3:**
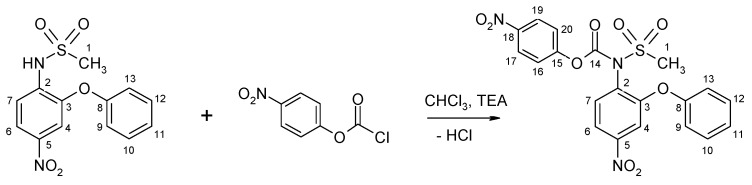
Reaction pathway of nimesulide derivatization with *p*-nitrophenyl chloroformate (NPCF) with atom numbering of **1**.

**Figure 2:**
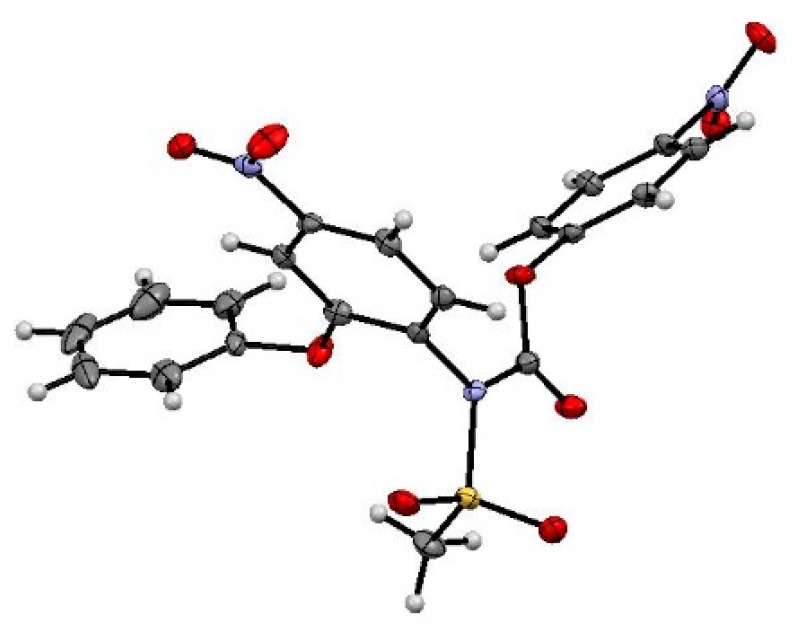
Schematic view of **1** (C—gray, N—blue, O—red, S—yellow). The crucial bond lengths and angles are S-C_1_ = 1.75; S-N = 1.69; N_1_-C_2_ = 1.45; N_1_-C_14_ = 1.38 Å, C_2_-N-S = 118.3(2) °, respectively (atom numbering according to Scheme 3 and deposited cif in CCDC). All bond lengths and torsion angles are collected in Table A1 (Appendix A).

Nimesulide was activated by obtaining the N-(4-nitrophenoxycarbonyl) derivative **1** from reaction of NPCF with **N**. Derivative **1** was fully characterized using NMR, mass spectrometry, and crystallography (Scheme 3). The schematic view of **1** is shown in Figure 2. 

The X-ray structure of **N** was reported in 1995 [17]. Significant changes in bond lengths occur around the central sulfonamide nitrogen upon replacement of the hydrogen atom by 4-nitrophenylcarbonate in **1**, namely elongation of the N-S bond from 1.64 to 1.69 Å, N-C_2_ bond from 1.41 to 1.45 Å, and S-C_1_ from 1.47 to 1.75 Å. The torsion angle S-N-C_2_ tightens from 124.7° in **N** to 118.3° in **1** due to steric hindrance of the 4-nitrophenylcarbonate substituent. We have noticed that **1** is stable in aqueous solution but reacts readily with amine groups on PAMAM G0.

**Scheme 4:**
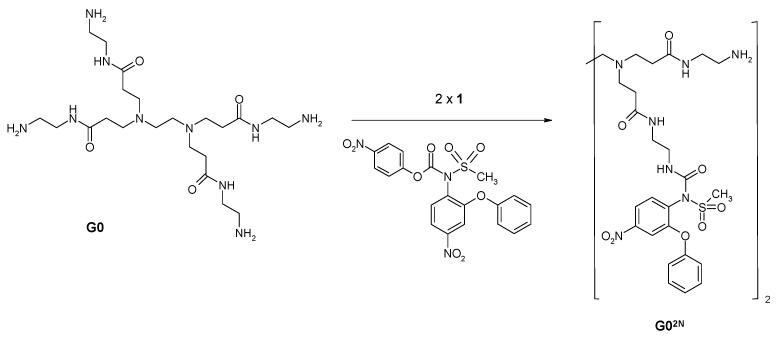
Synthesis of PAMAM G0 double substituted with **N** via carbonyl linker (**G0^2N^**).

**Figure A1:**
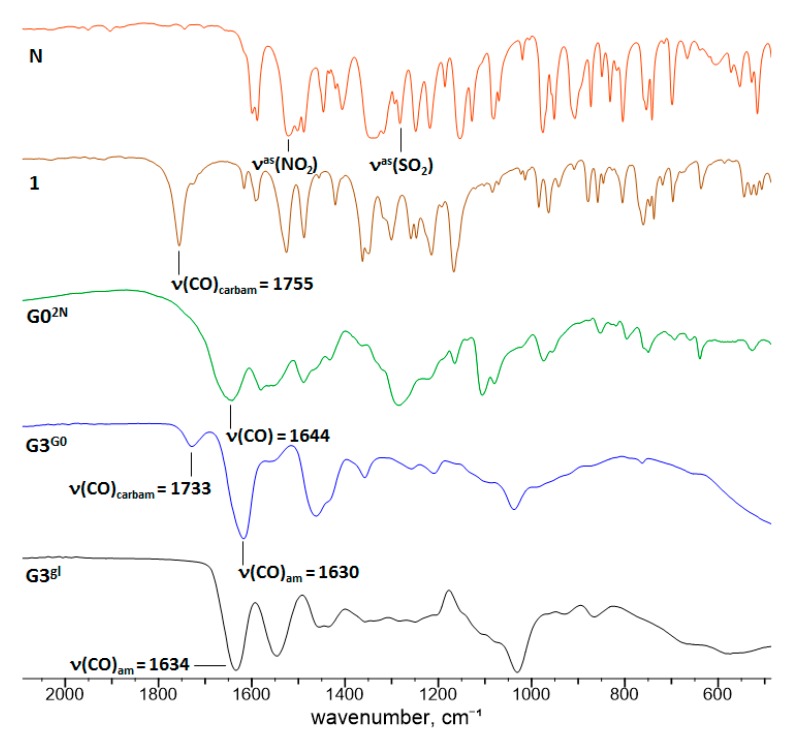
Infrared spectra (from top to bottom) **N** (red); **1** (brown) (both in KBr pellet); **G0^2N^** (green), **G3^G0^** (blue), **G3^gl^** (black); (all ATR).

Thus, **1** was used to covalently attach **N** via one-atom linker to PAMAM G0 to give G0 substituted with two molecules of **N** (Scheme 4). The urea bond formation was accompanied by release of 4-nitrophenol, which was removed by rinsing with chloroform. The characteristic IR carbamate ν(CO) band centered at 1755 cm^−1^ in **1** disappeared in **G0^2N^** and was replaced by a ν(CO) band centered at 1644 cm^−1^ from **G0** amide and a urea bond between **N** and **G0** (Figure A1). Obtained **G0^2N^** was further used to attach it to core **G3^gl^** after its activation with 14 equivalents of NPCF.

#### 2.1.3. Synthesis of Megamer with Conjugated G0

**Scheme 5:**
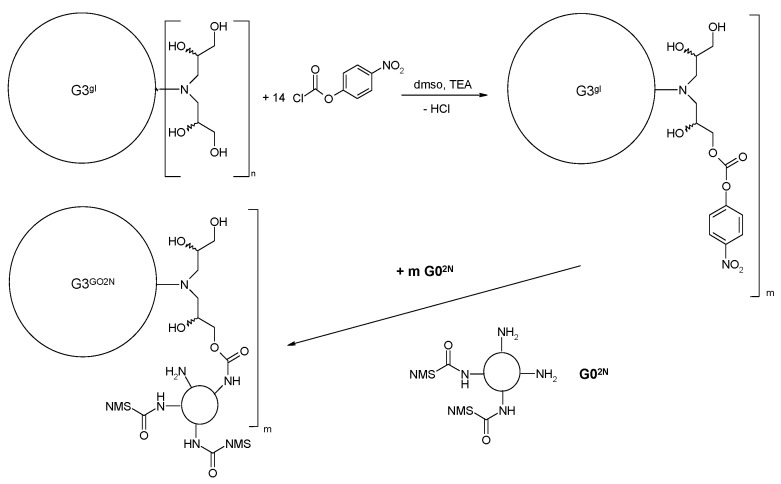
Synthesis scheme of megamer composed of **G3^gl^** core (*n* = 32), to which one-carbon linker is provided by reaction with *p*-nitrophenyl chloroformate (14 equivalents, *m* = 12). Here, NMS represents nimesulide.

**G3^gl^** was activated with 14 equivalents of NPCF in dry dimethylsulfoxide in the presence of excess TEA and used without isolation to bind 12 equivalents of **G0^2N^** (Scheme 5). 

**Figure 3:**
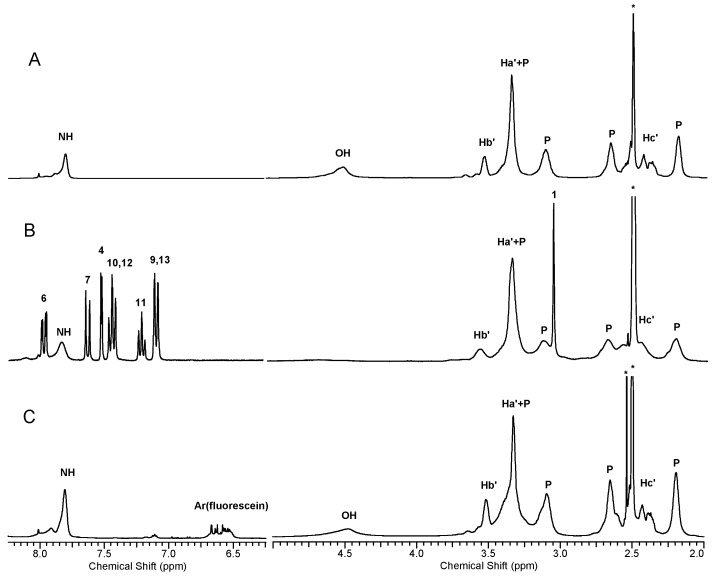
The relevant fragments of ^1^H NMR spectra of: **A**—**G3^gl^**, **B**—**G3^G02N^**, **C**—**G3^G0F^** in DMSO-d_6_. Intensity of resonances in the left trace B was multiplied by factor of 2. The resonances of nimesulide aromatic protons are labeled according to numbering shown in Scheme 3. The resonances from PAMAM G3 and PAMAM G0 cores are labeled P, while signals from 1,2-dihydroxypropyl substituents are labeled according to Scheme 2. Residual solvent and impurity resonances are labeled with asterisks.

The mixture was dialyzed extensively against water. The stoichiometry of the obtained megameric conjugate was determined by the ^1^H NMR spectrum of the product (Figure 3). Total integral intensity of CH resonances of **G3^gl^** corresponds to [804H] while intensities of OH and NH signals are [128H] and ca [60H], respectively (trace A). In the ^1^H NMR spectrum of **G3^G02N^**, the nimesulide aromatic proton resonance intensity (in the 8–7 ppm region) corresponds to [192H] (24 **N** attached) versus broad CH signals intensity [1308H] in the 4–2 ppm region (trace B). The core **G3^64gl^** contribution is [804H], while additional intensity derives from 12 **G0** ([432H]) and methyl groups of **N** ([72H]). The OH proton resonance is spread over the 4.25–5.25 ppm region and therefore integration of it is unreliable, as are NH broad resonances centered at 7.84 ppm. Thus, the average megameric conjugate bears 24 **N** prodrug units linked to **G0** via urea bonds, while **G0^2N^** subunits are linked to the **G3^gl^** core by carbamate bonds. IR spectra of the **G3^gl^** core show a strong carbonyl stretching vibration band ν(CO) centered at 1634 cm^−1^, while ν(CO) for the urea bond (maximum at 1644 cm^−1^) in **G0^2N^** is overlapped with that of amide ν(CO) of **G0**. The ν(CO) of the carbamate bond between **G3^gl^** and **G0** in megamers was observed at 1733 cm^−1^, well separated from other ν(CO) bands (Figure A1).

A similar synthesis of megameric conjugate was performed for FITC-labeled G0 (**G0^F^**). We deliberately used only four equivalents of NPCF to activate **G0^gl^** and 4 equivalents of **G0^F^** to obtain **G3^G0F^** for preliminary confocal microscopy studies on cell cultures. The ^1^H NMR spectrum of **G3^G0F^** (trace C) allowed us to determine the stoichiometry of this conjugate by integration of fluorescein aromatic resonances, which corresponds to [36H] (9H per one FITC) versus [948H] from **G3^gl^** core [804H] and 4 attached **G0** [144H]. 

#### 2.1.4. Molecular Weight and Size of Dendrimers and Megamers

The dispersity of molecular weight, molecular shape and size are important factors influencing the ability of the drug carrier to cross cell membranes by endocytosis. We determined the molecular weight of the obtained megamers, including the series of G2^gl^-G0, and G3^gl^-G0 synthesized in order to optimize the reagent stoichiometry in two-step synthesis followed by long purification of megamers by dialysis with water. The molecular weight and dispersity of megamers was determined using gel permeation chromatography (GPC) with dimethylformamide eluent. The column was calibrated with a series of G2–G5 dendrimers substituted with glycidol, for which we determined the average composition using ^1^H NMR spectra. Thus the reference compounds were very similar chemically to the dendrimers tested. 

**Figure A2:**
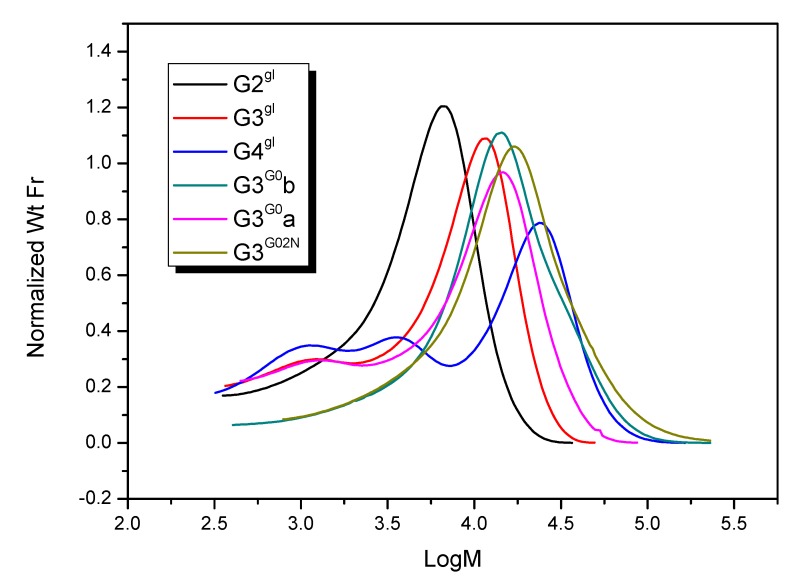
Molecular weight distribution curves of megamers G3^G0^ and G3^G02N^ estimated using GPC experiments using the references obtained by reaction of PAMAM dendrimers G2, G3, and G4 with gl according to the protocol described for **G3^gl^**. Obtained derivatives bear 46, 78, and 140 2,3-dihydroxyproyl substituents and averaged molecular weight 6600, 12,681, and 24,575 Da, respectively for G2^46gl^, G3^78gl^, and G4^140gl^. The stoichiometry of these G2^gl^, G3^gl^, and G4^gl^ derivatives was determined using ^1^H NMR spectroscopy. The G2^gl^, G3^gl^, and G4^gl^ were used as molecular weight standards to determine the molecular weight of megamers. The figure represents normalized weight fraction vs logM (where M—molecular weight). Samples of megamers G3^G0^a and G3^G0^b were obtained from G3^64gl^ : 18 NPCF : 20 G0 according to general protocol described in Experimental section. The average molecular weight of the samples G3^G0^b and G3^G0^a were estimated for conjugate before (b) and after (a) dialysis. Partial loss of G0 from the conjugate upon three days extensive dialysis can be concluded from that experiment (from average 13 to 10).

We have found that **G3^gh^**, upon reaction with 18 equivalents of NPCF followed by addition of 20 equivalents of **G0** to give **G3^G0^**, showed M_w_ corresponding to stoichiometry 1:13, while after prolonged dialysis of the same sample, the stoichiometry dropped to 1:10 (samples are named G3^G0^b before and G3^G0^a after dialysis, see Figure A2 and Table A2). Also using GPC, we found that G3:G0 stoichiometry in the **G3^G02N^** megamer was 1:9, which corresponds to 18 **N** equivalents per one megamer. This is very similar stoichiometry to that obtained from integration of resonances of **N** in ^1^H NMR spectrum of **G3^G02N^** megamer (*vide supra*). Thus the prolonged dialytic purification of megamers with water resulted in ca 10–25% loss of G0 from a megamer.

The molecular size of the megamers was estimated using the DLS method and compared to a series of GM^gl^ dendrimers synthesized to serve as a reference series, in addition to known GM dendrimers (where M is generation 2-5). The DLS-estimated diameters for dendrimers and megamer (determined in volume mode) are collected in Table A3. The **G3^G02N^** megamer diameter was 5.07 ± 0.14 nm, which is similar to that of G4^gl^ (5.12 nm). 

#### 2.1.5. Structural and Height Analysis of Dendrimers Using AFM 

**Figure 4:**
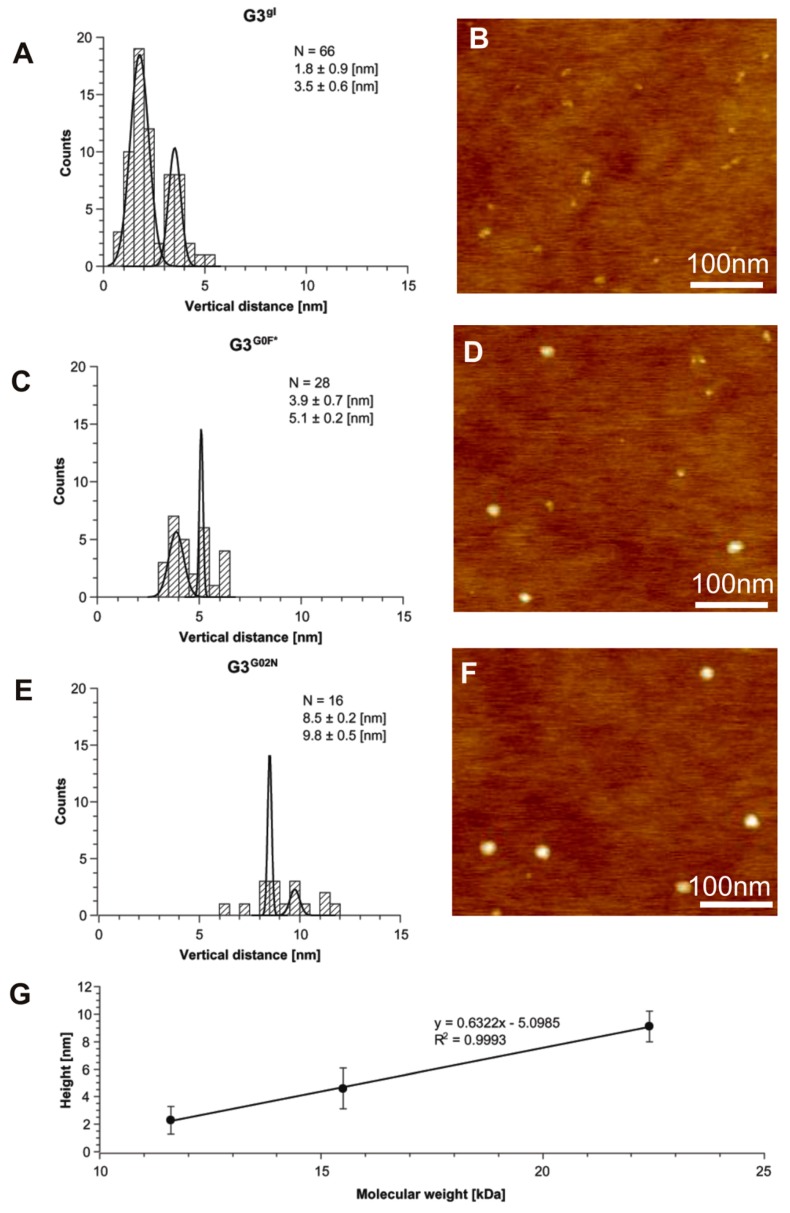
AFM images with height diagrams of dendrimers: (**A**,**B**) **G3^gl^**; (**C**,**D**) **G3^G0F^**; (**E**,**F**) **G3^G02N^**; (**G**) Linear relationship of the dendrimer’s height as a function of its molecular weight with high correlation coefficient (R^2^ = 0.9993).

The height of dendrimers **G3^gl^**, **G3^G0F^** and **G3^G02N^** characterized using AFM is presented in Figure 4. In the case of **G3^gl^**, two populations are observed with heights 1.8 ± 0.9 nm and 3.5 ± 0.6 nm (*n* = 66), respectively (Figure 4A). For **G3^G0F^**, two populations are observed with heights 3.8 ± 1.3 nm and 5.4 ± 0.2 nm (*n* = 28) (Figure 4C). In the case of conjugate **G3^G02N^**, populations with the heights 8.5 ± 0.2 nm and 9.8 ± 0.5 nm (*n* = 16) (Figure 4E) were observed. 

A linear relationship was found by plotting the measured height of dendrimers as a function of molecular weight with high correlation (R^2^ = 0.9993) (Figure 4F). To determine the distribution of acquired numerical data, histograms were plotted and a Jarque‒Bera test was performed. None of data series describing vertical distance of dendrimers comes from a normal distribution (*p*-value < 0.05). The set of vertical distance data was also tested for the presence of outliers by means of a modified Thompson tau test. For all dendrimers, data outliers were found at the 95% confidence level. Occurrence of outliers may be evidence of gradual aggregation of dendrimers, which take the form of larger objects on the mica surface.

### 2.2. Biology

#### 2.2.1. Cytotoxicity

**Figure 5:**
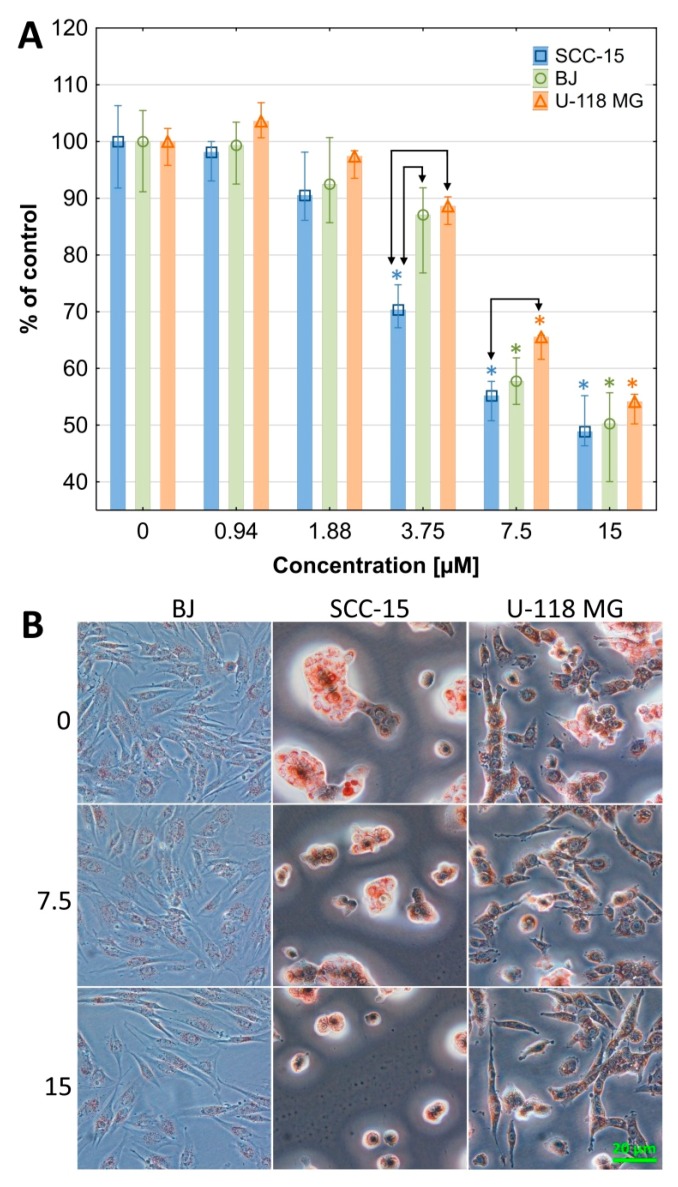
**G3^G02N^** cytotoxicity against BJ, SCC-15 and U-118 MG cells after 24 h treatment. (**A**) Results are presented as median of triplicate assays from three independent experiments, expressed as a % of non-treated controls. The whiskers are the lower (25%) and upper (75%) quartile ranges. * *p* < 0.05; Kruskal–Wallis test (against non-treated respective control). (**B**) Changes in cell morphology after 24 h treatment with **G3^G02N^** and after 1 h incubation with neutral red. Red vesicles are lysosomes containing dye. Numbers indicate a micromolar concentration of **G3^G0N^**. Images were obtained with a contrast phase microscope (Olympus IX-83).

To evaluate the utility of synthesized megameric conjugate **G3^G0N^** containing the cyclooxygenase-2 (COX-2) inhibitor nimesulide, three human cell lines were chosen: squamous carcinoma (SCC-15) and glioblastoma (U-118 MG), both overexpressing COX-2, and comparative normal human skin fibroblasts (BJ) with lower expression of COX-2 [3]. The cytotoxicity assay revealed inhibitory action of the studied conjugate at low 7.5 µM concentration and, in the case of squamous carcinoma cells (SCC-15), even as low as 3.75 µM, after 24 h of exposure. At the highest concentration (15 µM) all cell lines revealed about 50% decrease in viability. The most resistant were glioma cells and the least SCC-15 cells (Figure 5). Over the entire concentration range, the viability of squamous cell carcinomas was noticeably lower than other cell lines, particularly glioma cells. Statistical analysis revealed, however, that significant differences appeared at concentrations of 3.75 and 7.5 µM. Viability of normal fibroblasts had an intermediate degree. Microscope images collected during the assay performance confirmed obtained results. Changes in cell morphology were seen as concentration dependent cell shrinkage and decrease in cell number, cellular protrusion size and neutral red content in the lysosomes. The described symptoms of cell degradation were the most visible in SCC-15 cells. The biological effect of **G3^G02N^** appeared probably from the presence of **N** on the **G3^G02N^** surface. As described in many papers, **N**, a preferable COX-2 inhibitor, is a promising chemopreventive and antineoplastic agent that acts via blocking COX-2 and decreasing the concentration of prostaglandins inside the tumor or via other COX-independent pathways [18,19]. It was assumed that SCC-15 and U-118 MG cells with elevated COX-2 levels [3] would be more sensitive to a megamer containing **N**. SCC-15 cells showed a significant stronger decrease of viability at 3.75 µM than normal cells with a lower COX-2 level. However, glioma cells did not react more strongly than BJ cells. Slightly more light is shed on this problem by our recent studies, where we showed that the action of nimesulide-substituted PAMAM G3 dendrimers selectively reduced the viability of SCC-15 cells compared to normal fibroblasts. However, this effect was not based on the COX-2 and PGE_2_ axis [4]. Therefore, it should be assumed that the action of the nimesulide substituted PAMAM dendrimers, including the tested megamer, can be also realized via independent COX-2 and PGE_2_ pathways and requires further, profound studies of this issue.

Moreover, both PAMAM G0 and G3 dendrimers showed much lower toxicity to the cancer cells compared to obtained **G3^G02N^**. G0 PAMAM dendrimers were not toxic against colorectal adenocarcinoma (Caco-2) cells up to 10 mM concentration after 3 h of incubation [20]. In addition, Zeng et al. demonstrated lack of G0 PAMAM dendrimer toxicity in human neural progenitor cells (hNPCs) up to 200 µM concentration after 24 or even 72 h of incubation [21]. The PAMAM G3 IC_50_ for normal human fibroblasts (BJ) after 24 h incubation was higher than for SCC-15 cells (7.5 and 30 µM, respectively) [22]. Attachment of the PAMAM G0 dendrimer molecules with **N** to the **G3^gl^** dendrimer resulted in a reversal of the biological effect on squamous carcinoma cells, which became more sensitive than normal human fibroblasts.

#### 2.2.2. Cellular Accumulation

**Figure 6:**
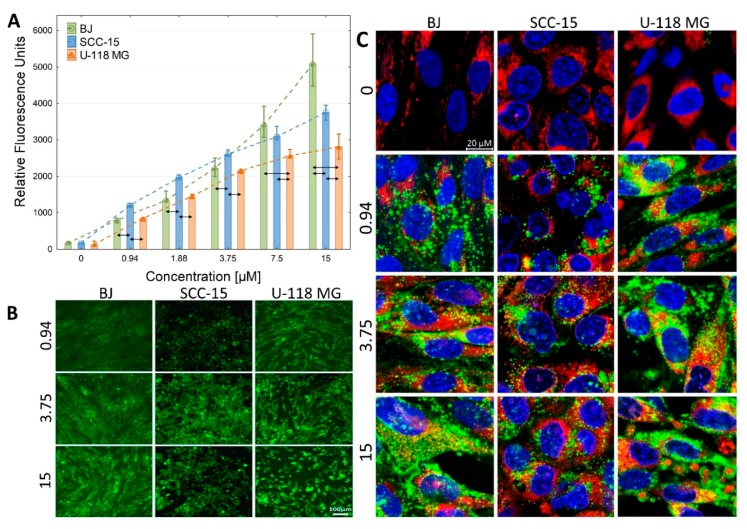
(**A**) Dose–dependent cellular accumulation of G3^G02N^* in BJ, SCC-15 and U-118 MG cells after 24 h incubation. Results are presented as a median of triplicate assays from three independent experiments, expressed as a relative fluorescence unit per 2 × 10^4^ cells. The whiskers are lower (25%) and upper (75%) quartile ranges. The arrows indicate differences in fluorescence between cell lines at appropriate concentrations, estimated with the Mann‒Whitney U test (*p* < 0.05). (**B**) Images present accumulation of G3^G02N^* (green signal) after 24 h incubation obtained with a fluorescence microscope (Olympus IX-83). (**C**) Representative images from confocal microscopy showing degree of penetration of fluorescently labelled megamer (green signal) into DAPI labelled nuclei (blue signal) or MitoTracker labelled mitochondria (red signal). Micromolar concentrations of G3^G02N^* are indicated.

Fluorescent labeled megamer (G3^G02N^*) penetrated into all three cell lines efficiently, but the profile of its cellular accumulation differed in particular cell types. The most efficient penetration of G3^G02N^* was observed in SCC-15 cells at a concentration of 3.75 µM, similar to that in BJ and somewhat lower than in U-118 MG glioma cells (Figure 6A,B). 

At 7.5 and 15 µM concentrations, the most efficient uptake was found in normal fibroblast cells, followed by SCC-15 and U-118 MG cells. The reason for this trend could be that BJ cells are more resistant to the toxic effects of **G3^G02N^** and maintained a high degree of intracellular nanoparticle transport. Furthermore, phagocytic activity of these cells may be contributing to this phenomenon [23]. The lower accumulation of G3^G02N^* in glioblastoma and SCC-15 cells may be caused by active xenobiotic efflux systems present in cancer cells, including gliomas [24].

The degree of fluorescently labelled megamer accumulation in mitochondria, nuclei or in other subcellular organelles after 24 h incubation were estimated with confocal microscopy by determining the degree of co-localization with fluorescently stained organelles. Studies revealed that G3^G02N*^ was present in intracellular compartments of all studied cell lines (Figure 6C). Most of the megamer absorbed by the cells remained in endocytic vesicles and in lysosomal vesicles. A significant part of the dendrimer was also dispersed in the cytoplasm of the cells, especially at higher concentrations of G3^G02N*^. Only a small part accumulated in the mitochondria in a concentration dependent manner. In addition, G3^G02N*^ penetrated the mitochondria of normal fibroblasts and glioma cells to a larger degree than squamous carcinoma cells at all concentrations (Figure 6C, yellow signal). The penetration of the megamer into the nuclei was negligible and only noticeable in the case of normal fibroblast cells at the highest concentration of 15 μM (Figure 6C, light blue signal). Our findings are in agreement with the observations of others concerning PAMAM dendrimers [25,26]. These results confirm that the studied megamer, in addition to its anticancer properties, can be a proper agent to deliver drugs into resistant cancer cells.

#### 2.2.3. Anti-Proliferation

In this study, the megamer substituted with nimesulide **G3^G02N^** was synthesized as a highly promising anti-cancer agent due to its anti-proliferative properties. **N** exert a strong anti-proliferative effect by promoting cell cycle arrest in multiple gastric cancer cell lines [27]. Moreover, the anti-proliferative action of this highly preferable COX-2 inhibitor was demonstrated against non-small cell lung cancer, hepatoma SMMC-7721 cells, human gastric adenocarcinoma SGC7901 cells, human pancreatic cancer cells and human esophageal adenocarcinoma OE33 cells [18,28].

**Figure 7:**
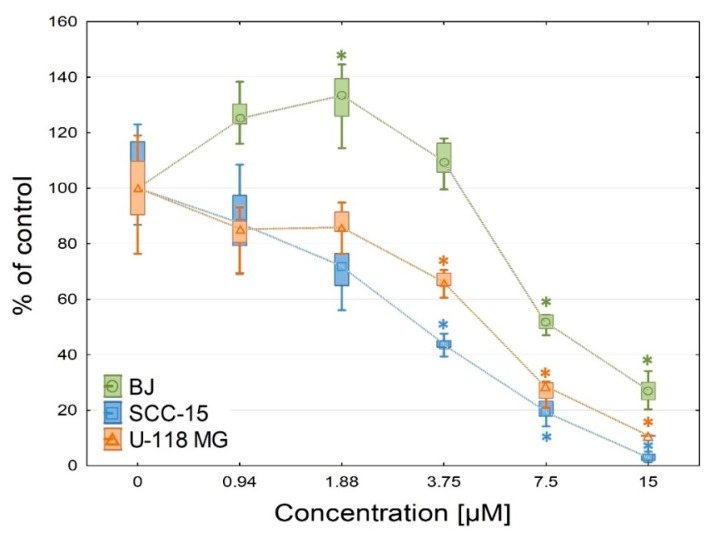
Influence of **G3^G02N^** on BJ, SCC-15 and U-118 MG cell proliferation after 72 h incubation, estimated after DAPI staining. Data are presented as % of non-treated control. Results are a median of triplicates from three independent experiments. The whiskers are lower (25%) and upper (75%) quartile ranges. * indicate statistically significant differences estimated with the Kruskal‒Wallis test (*P* < 0.05).

In this study, **G3^G02N^** revealed strongly selective, antiproliferative action against cancer cells compared to normal fibroblasts after 72 h incubation (Figure 7). 

The inhibition of cell division was particularly evident in the SCC-15 line and only slightly weaker in the case of glioblastoma cells, with IC_50_ values of 3.19 and 4.48 µM, respectively, compared to 9.28 µM for normal human fibroblasts. Likewise, the strong inhibitory action of nimesulide after 72 h incubation against human squamous carcinoma cells (A431) was observed by Khodaie et al. with IC_50_ = 250 µM [29]. Differences in IC_50_ values for native drug and **G3^G02N^** suggest that the decrease of cell proliferation is affected not only by nimesulide itself but also additionally by its megameric carrier. It has been noticed that proliferation of normal BJ cells grows to 134% at 1.88 µM concentration. Similar cell growth enhancement was observed for PAMAM G2, G4, and G6 neat dendrimers in HeLa and HEK293T lines at 100–500 nM concentration of dendrimer [30].

## 3. Materials and Methods 

### 3.1. Materials

PAMAM dendrimers were synthesized starting from ethylenediamine according to a modified protocol described by Tomalia [31]. All reagents for chemical syntheses, i.e., glycidol (mixture of enantiomers), methyl acrylate, ethylenediamine, and solvents, were purchased from Sigma-Aldrich (St Louis, Missouri, USA) as reagent grade and used as received. For biological studies, Eagle’s Minimum Essential Medium (EMEM), Dulbecco’s Modified Eagle’s Medium (DMEM and DMEM: F-12), fetal bovine serum (FBS), penicillin and streptomycin solution were obtained from ATCC (Manassas, VA, USA). Trypsin-EDTA solution, phosphate-buffered saline (PBS) with and without magnesium and calcium ions, 0.4% trypan blue solution, fluorescent marker DAPI (4′,6-diamidino-2-phenylindole, dihydrochloride) were purchased from Thermo Fischer Scientific (Waltham, Massachusetts, USA). Hydrocortisone, 0.33% neutral red solution (3-amino-6-dimethylamino-2-methyl-phenazine hydrochloride), was obtained from Sigma-Aldrich (St Louis, Missouri, USA). Cell culture dishes were from Corning Incorporated (Corning, NY, USA) or Nunc (Roskilde, Denmark).

### 3.2. Syntheses

#### 3.2.1. Synthesis of PAMAM G0 Dendrimer Substituted with 8 Glycidol Molecules, G0^gl^

Glycidol (**gl**, 0.9 mL, 0.967 g, 13.0 mmol) was added dropwise to a solution of PAMAM G0 (0.884 g, 1.63 mmol) in 20 mL of methanol with magnetic stirring. The mixture was left at room temperature for 2 days. Then methanol and excess **gl** were removed by vacuum rotary evaporation. Yellow syrup was obtained, which was identified using ^1^H NMR as **G0^gl^** (Figure 1).

^1^H-NMR (DMSO-*d*_6_; for atom numbering see Scheme 1): chemical shift [ppm] (intensity, multiplicity, assignment): 7.83 ([4H], bs, H-6’); 4.47 ([16H], bs, OH); 3.51 ppm ([8H], q, H_b’_); 3.22 ppm ([16H], m, H_a’_); 3.09 ppm ([8H], H-7’); 2.63 ppm ([8H], t, H-2’); 2.51 ppm ([8H], m, H-8’); 2.42 ppm ([4H], s, H-1’); 2.33–2.40 ppm ([16H], m, H_c’_); 2.18 ppm ([8H], t, H-4’).

#### 3.2.2. Synthesis of PAMAM G3 Dendrimer Substituted with 64 Glycidol Molecules (G3^gl^): The Megamer Macromolecular Core 

Dendrimer PAMAM G3 in methanol (525 mg, 0.076 mmol in 5 mL; 15.2 mM solution) was added dropwise into a 25 mL round bottom flask containing glycidol (0.35 mL, 374 mg, 5.10 mmol) in 2 mL of methanol. The mixture was left at room temperature for 2 days. After two days, the solution was transferred into a nitrocellulose dialytic bag (MW_cutoff_ = 3 kDa) and dialyzed against water for three days. Water was removed under reduced pressure by rotary evaporation and the resulting solid residue was dried under 0.2 mm Hg pressure overnight. A 90% yield (810 mg) of **G3gl** was obtained as a brown syrup and was characterized using ^1^H NMR spectroscopy. The isolated **G3^gl^** macromolecular core was found to be very soluble in water and in dimethyl sulfoxide. 

^1^H-NMR (DMSO-*d*_6_; for atom numbering see Scheme 2): chemical shift [ppm] (intensity, multiplicity, assignment): 7.80 ([60H], bs NH); 4.47 ([128H], bs, OH); 3.51 ([64H], bs, H_b’_); 3.33 ([128H] H_a’_ + [120H] PAMAM); 3.10 ([120H], bs, PAMAM); 2.65 ([120H], bs, PAMAM); 2.30–2.45 ([128H], m, H_c’_ + [4H], s, PAMAM); 2.20 ([120H], bs, PAMAM).

IR (Figure A1, Appendix A): ν(CO) = 1634 cm^−1^. Theoretical molecular weight = 11 645 Da.

#### 3.2.3. Synthesis of N-(4-Nitrophenoxycarbonyl) Nimesulid: N-(4-Nitrophenoxycarbonyl), N-(4-Nitro-2-Phenoxyphenyl) Methanesulfonamide, 1

Nimesulide (220 mg, 0.713 mmole) was dissolved in chloroform (5 mL) and triethylamine (TEA, 0.4 mL). Then 4-nitrophenylchloroformate was added (288 mg, 1.43 mmoles) in portions with continuous stirring and the mixture was left under nitrogen at room temperature for 24 hrs. The chloroform layer was washed three times with water (25 mL) and the solvent was removed under reduced pressure. The mixture was chromatographed with chloroform: ethyl acetate (7:1) on silica gel. **1** was eluted as first fraction as identified using NMR and MS. Crystals of **1** were grown in an NMR tube upon layering the solution (0.05 M) with hexane. The percent yield of **1** was 52.9% (250 mg).

Analytical data (for atom numbering see Scheme 3):

^1^H NMR (CDCl_3_): 8.25 (d, J_16–17_ = 9.2 Hz, [2H], H-17,19); 8.04 (dd, J_6–7_ = 8.7 Hz, J_6–4_ = 2.5 Hz, [1H], H-6); 7.70 (d, [1H], H-7); 7.69 (d, 1H], H-4); 7.46 (t, [1H], H-10,12); 7.32 (t, J_11-10_ = 7.3 Hz, [1H], H-11); 7.27 (d, [2H], H-16,20); 7.10 (d, J_9-10_ = 7.5 Hz, [2H], H-9,13); 3.54 (s, [3H], H-1).

^13^C NMR (CDCl_3_): 154.7 (C-5); 154.2 (C-18 and C-8); 149.7 (C-14); 149.4 (C-3); 146.0 (C-15); 132.9 (C-6); 131.0 (C-10,12); 130.4 (C-2); 126.4 (C-11); 125.4 (C-17,19); 122.1 (C-16,20); 119.8 (C-9,13); 118.2 (C-7); 112.6 (C-4); 41.9 (C-1). 

AuNPET LDI MS: 396.07 (90%, Au^+^), 237.50 (30%, [**1** + 2H]^2+^); 393.93 (100%, Au_2_^+^); 496.04 [25%, **1** + Na]^+^; 590.90 (60%, Au_3_^+^). Theoretical molecular mass for C_20_N_3_O_9_SH_15_-473.05.

IR (in KBr): **1**: ν(CO) = 1756 cm^−1^; ν_as_(NO_2_) = 1526 cm^−1^; ν_as_(SO_2_) = 1301 cm^−1^, for comparison **N**: ν_as_(NO_2_) = 1522 cm^−1^; ν_as_(SO_2_) = 1293 cm^−1^.

#### 3.2.4. Synthesis of PAMAM G0-Bis-Carbonylnimesulide (G0^2N^) and Single Fluorescein-Labeled PAMAM G0 (G0^F^)

150 mg (0.32 mmol) of **1** in chloroform (1 mL) was added to PAMAM G0 (83 mg, 0.16 mmol in 4 mL methanol) at room temperature with vigorous stirring for 24 h. Then the solution was heated to reflux for one hour and solvents were evaporated in vacuo. **G0^2N^** was rinsed with chloroform and isolated as pure compound in 35% yield. 

^1^H NMR (DMSO-*d*_6_): 8.14 (bs, [2H], NH(G0); 7.80 (dd, [2H], H-6); 7.47 (d, J_6–4_ = 2.8 Hz, [2H], H-4); 7.31 (d, J_6–7_ = 9.4 Hz, [2H], H-7); 7.28 (t, [4H], H-10,12); 6.99 (t, J_10–11_ = 7.3 Hz, [2H], H-11); 6.84 (d, J_9–10_ = 7.9 Hz, [4H], H-9,13); 6.02 and 5.89 (both bs, [1H], NH); 3.13–2.98 (bm, [12H], G0); 2.62 (bm, [12H], G0); 2.58 (s, [6H], CH_3_-1); 2.41 (s, [4H], G0); 2.19 (t, [8H], G0, CH_2_-CO-). IR (Figure A1): ν(CO) = 1644 cm^−1^.

Fluorescein labeled G0 was synthesized on a 0.10 mmolar scale. To the solution of 51.7 mg (100 µmoles) PAMAM G0 in methanol (2 mL), fluorescein isothiocyanate (FITC, 38.9 mg, 100 µmoles in 2 mL methanol) was added stepwise with vigorous stirring. The red precipitate of **G0^F^** was collected using filtration, washed with methanol and dried in vacuo.

^1^H NMR (DMSO-*d*_6_): 8.50–7.94 (bm, [5H], NH); 7.17–6.25 (m, [9H], ar(F)); 3.53 (bs, [2H], G0); 3.23 (bs, [2H], G0); 3.09 (bs, [6H], G0); 2.70–2.50 (overlapped m, [14H], G0); 2.34 (bs, [4H], G0); 2.24–2.00 (overlapped m, [8H], G0). IR (Figure A1): ν(CO) = 1634 cm^−1^.

#### 3.2.5. Synthesis of megamers G3^gl^-12G0^2N^ and G3^gl^-4G0^F^

**G3^G02N^**: 100 mg **G3^gl^** (8.5 µmoles) was dissolved in 2 mL of DMSO and 0.5 mL of TEA. To this solution, solid NPCF (28.2 mg, 102 µmoles) was added with vigorous stirring. The obtained mixture was immediately used to react with 119 mg (100 µmoles) of **G0^2N^** in 2 mL DMSO at room temperature overnight. Then the mixture was dialyzed in nitrocellulose tubing (MW_cutoff_ = 3 kDa) against water for 4 days. Water was removed under reduced pressure and the ^1^H NMR spectrum was recorded. Based upon integral intensity of **N** versus PAMAM resonances the stoichiometry of conjugate was determined to be **G3^gl^–12G0^2N^** (see Figure 2).

^1^H NMR (DMSO-*d*_6_): **N** resonances: 7.98 (dd, J_6–7_ = 8.8 Hz, J_6–4_ = 2.5 Hz, [24H], H-6); 7.63 (d, [24H], H-7); 7.52 (d, [24H], H-4); 7.44 (t, [48H], H-10,12); 7.21 (t, J_11–10_ = 6.4 Hz, [24H], H-11); 7.09 (d, J_9–10_ = 7.1 Hz, [48H], H-9,13); 3.05 (s, [72H], H-1), PAMAM **G3^gl^** + **G0** resonances: 3.53 (bs, [64H], H_b’_); 3.33, 3.12, 2.97, 2.85, 2.67, 2.57, 2.45, 2.21 (unresolved broad resonances, ca [1200H], theor. [1172H] with contribution from G3 [484H], H_a’_ and H_c’_ of 64 **gl** [256H], and 12 G0 [432H]).

The weight-averaged molecular weight M_W_ by GPC was ca 22.4 kDa with 1.63 M_w_/M_n_ dispersity (and 37 kDa from M_Z_) vs theoretical average molecular weight 26,249 kDa. 

The **G3^G02N^** conjugate was tested for toxicity against cancer cells. Additionally, ca 1 µmol of **G3^G02N^** (26 mg) was labeled with one equivalent of FITC and used for confocal microscopic monitoring in cell lines (G3^G02N^*, vide infra).

**G3^G0F^**: 31 mg **G3^gl^** (2.7 µmoles) was dissolved in 1.5 mL of DMSO and 0.3 mL of TEA. To this solution, solid NPCF (2.3 mg, 11.4 µmoles) was added with vigorous stirring. Then, 0.115 mL of 0.10 M **G0^F^** solution in DMSO (11.5 µmoles) was added and the mixture left for 24 h at room temperature. Then the mixture was dialyzed against water for 3 days in the dark. The solvents were removed under reduced pressure, yielding 35 mg of **G3^gl^**. **G3^gl^** was conjugated with 4 equivalents of **G0^F^** based upon integral intensity of **F** aromatic resonances versus total intensity of **G3** and **G0** resonances. The estimated yield was 84%. The average molecular weight determined using GPC was ca 15.5 kDa vs theoretical molecular weight 15,383 for G3–4G0^F^ stoichiometry. The aqueous solution of **G3^G0F^** was used to deposit the monomolecular layer in mica to estimate molecular size using AFM (vide infra).

^1^H NMR (DMSO-*d*_6_): 7.91 + 7.82 (bs, bs, ca [70H], NH); 6.71–6.50 (unresolved multiplets, [36H], aromatic CH from fluorescein); 4.47 (bs, ca [110H], OH); PAMAM **G3^gl^** + **G0** resonances: 3.57–2.13 (total integrity ca [950H] versus theoretical [938H] with contribution from **G3^gl^** [824H] and 4 **G0** [144H].

### 3.3. Cell Cultures

Human squamous cell carcinoma (SCC-15) and human glioblastoma (U-118 MG) cell lines obtained from ATCC (Manassas, VA, USA) were cultured in DMEM (doubling time 48 and 35 h, respectively). Normal fibroblast (BJ) purchased from ATCC (doubling time 1.9 days) were grown in EMEM. Each medium was supplemented with 10% heat-inactivated FBS and 100 U/mL penicillin, and 100 μg/mL streptomycin. Cells were cultured as described [22]. All biological tests were carried out in triplicates in three independent experiments.

#### 3.3.1. Cytotoxicity Neutral Red Assay (NR)

BJ, SCC-15 and U-118 MG cells were seeded in flat-bottom 96-well culture plates in triplicates at a density of 1 × 10^4^ cells/well. After 24 h, working solutions of **G3^G02N^** were prepared (0.94–15 µM) in the corresponding culture media and added to cells (100 μL/well). The DMSO concentration was adjusted to 0.4% in all samples, which had no significant effect on treated cell lines. Following a 24 h incubation, an NR assay was performed as described before [32].

#### 3.3.2. Cellular Accumulation of Megamer

Cells were seeded in 96-well plates at a density of 2 × 10^4^ cells/well (BJ and U-118 MG) or 4 × 10^4^ (SCC-15). After 24 h incubation cells were treated with G3^G02N^* in the range of concentration 0–15 µM in complete medium for 24 h. After incubation plates were centrifuged (2000 rpm, 5 min), cells were washed with PBS and fixed with 3.7% formaldehyde. Then, 600 nM DAPI solution in PBS was added (100 µL/well) and incubated for 1 h at room temperature. Fluorescence signals were read at 485/530 nm for FITC and 360/460 nm for DAPI with an Infinite M200 PRO Microplate Reader (TECAN Group Ltd., Switzerland). The DAPI staining was used to estimate the number of cells and calculate fluorescence signals per equal cell number.

#### 3.3.3. Confocal Microscopy

Cells were cultured on microscope chamber slides (Nunc, Denmark) for 48 h at a density of 60 × 10^4^ or 1.2 × 10^5^ cells (for BJ and U-118 MG or SCC-15 cells, respectively) in 400 μL of complete medium. FITC-labeled megamer was added at 0 to 15 μM concentrations (400 μL/well). After 24 h of incubation and washing (2×PBS), the cells were fixed with 3.7% formaldehyde for 15 min and stained with 600 nM DAPI solution in PBS (1 h, RT). Images from each well were collected using a confocal microscope (Olympus FV10i, Tokyo, Japan) at 488/530 nm for FITC, 405/461 nm for DAPI, and 644/665 nm for MitoTracker. Images were collected in the Z-axis position at the largest nuclear cross section area. Pinhole was set for 1 AU (airy unit) and the obtained images had an optical section thickness of approximately 1.02 μm. Image processing was performed with the ImageJ software (Bethesda, MD, USA).

#### 3.3.4. Proliferation Assay

For assay, cells were seeded into 96-well microplates at a density of 5 × 10^3^ cells/well and incubated for 24 h at 37 °C. After medium removal, **G3^G02N^** solutions were prepared as described above in medium (200 µL/well). The plates were then incubated for 72 h. Then, plates were centrifuged (2000 rpm, 5 min). Cells were then washed with PBS and fixed in 3.7% formaldehyde solution in PBS and stained with 600 nM DAPI solution in PBS (100 µL/well, 1 h). The fluorescent signal, proportional to the number of cells, was measured in a Tecan Infinite M200 PRO Multimode Microplate Reader (TECAN Group Ltd., Switzerland) at 360/460 nm. The results were expressed as % of the control (DMSO treated cells).

### 3.4. Methods

#### 3.4.1. Spectroscopy

1-D ^1^H, ^13^C NMR as well as 2-D ^1^H-^1^H COSY and ^1^H-^13^C HSQC and HMBC spectra were recorded with Bruker 300 MHz instrument. Gold enhanced target laser desorption ionization mass spectra were recorded with a Bruker Autoflex Speed reflectron time-of-flight mass spectrometer equipped with a SmartBeam II laser (352 nm) in 80–2080 *m/z* range [33].

IR spectra were taken with ALPHA FT-IR Bruker instrument in KBr pellets and ATR mode.

#### 3.4.2. Atomic Force Microscopy Studies

AFM images were recorded in air with a Nanoscope IIId scanning probe microscope with Extender Module (Bruker). Standard tapping mode AFM probes (NanoAndMore, Watsonville, California, USA) were used with a resonance frequency in the range of 200–400 kHz, with a typical spring constant of 42 N/m and with a nominal apex radius of silicon tip curvature around 7 nm. The samples with dendrimers were placed on freshly cleaved ultra-clean mica (Nano and More) and incubated at room temperature for 60 sec. The mica discs were then rinsed with purified 18.2 MΩ deionized water and dried using gentle nitrogen gas flow. All samples were measured at room temperature in air. Structural analysis and height measurements of acquired images were performed with Nanoscope v.6.13 software (Watsonville, California, USA).

#### 3.4.3. Molecular Weight Estimation with Gel Permeation Chromatography 

The average molecular masses, *M*n, *M*w and dispersity *M*w/*M*n of the products were measured using gel permeation chromatography (GPC) using a RI detector (Shodex RI-71). The GPC instrument was equipped with TSKgel GMH_HR_-M and TSKgel GMH_HR_ Guard column packed with styrene divinylbenzene-type gel. The measurements were performed at a temperature of 22 °C. All samples were dissolved in N,N-dimethylformamide (HPLC grade) containing 5 mmol/L LiCl. The flow rate of the carrier solvent was 1.00 mL/min. The sample injection volume was 100 μL. The average molecular masses and dispersity were determined using OmniSEC software (Dublin, Ireland). The G2, G3, and G4 PAMAM dendrimers bearing 46, 78, and 140 2,3-dihydroxyproyl substituents and averaged molecular weight: 6600, 12,681, and 24,575 Da, respectively for G2^46gl^, G3^78gl^, and G4^140gl^ were used for calibration. For details see Appendix A: Figure A2 and Table A2.

#### 3.4.4. Dynamic Light Scattering Measurements

**Figure A3:**
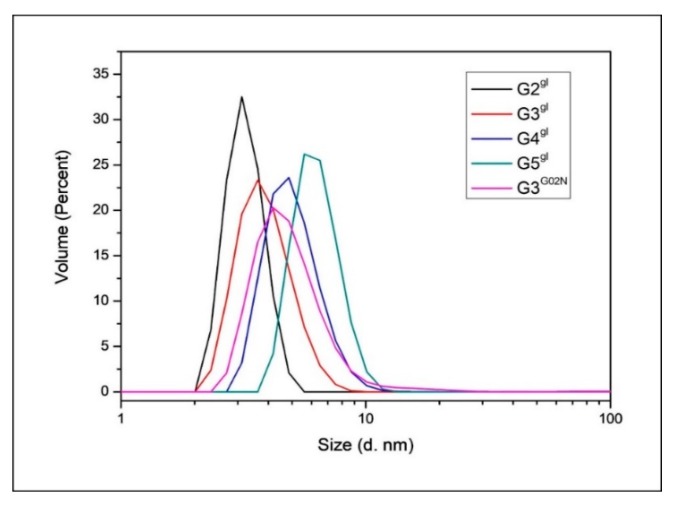
Size distribution of **GM^gl^** dendrimers and **G3^G02N^** megamer (M—dendrimer generation). The PAMAM dendrimers coated with 2,3-dihydroxypropyl substituents were synthesized for these studies. The composition of G2^gl^–G5^gl^ was determined using ^1^ H NMR spectroscopy. The NMR studies on the series will be reported separately.

DLS measurements were performed using a Zetasizer nano ZS instrument. Measurements were made under back-scattering conditions (fixed scattering angle 173°). All dendrimers were dissolved in water at ca 1.0 mM concentration. The size of dendrimers and megamers were calculated in volume mode. The results are presented graphically (Figure A3) and values of diameter [nm] are collected in Table A3.

#### 3.4.5. Crystallographic Measurements 

X-ray diffraction data of **1** was collected on an Xcalibur diffractometer with Saphire detector (Mo-Kα radiation; λ = 0.71073 Å) at 100 K. Data reduction and analysis were carried out with the CrysAlis program [34]. Structure was solved by direct methods using the SHELXS program and refined using all *F*^2^ data, as implemented by the SHELXL program [35]. Non-hydrogen atoms were refined with anisotropic displacement parameters. All H atoms were placed at calculated positions, and before the last cycle of refinement all H atoms were fixed and were allowed to ride on their parent atoms. 

2(C_20_H_15_N_3_O_9_S), CHCl_3_, triclinic, *P*-1, a = 10.583(3) Å, b = 12.774(3) Å, c = 17.974(3) Å, α = 72.60(3)°, β = 75.67(2)°, γ = 87.64(2)°, V = 2245.1(10) Å^3^, T = 100(2) K, R = 0.062, wR = 0.123 [5197 reflections with I > 2σ(I)] for 631 variables.

The .cif file has been deposited in CCDC, number 1910753, and is available upon request via www.ccdc.cam.ac.uk.data/data_request.cif.

## 4. Conclusions

PAMAM G3 and G0 megamer composed of PAMAM G3 dendrimer with 64 terminal propyldiol substituents serving as a core and 12 PAMAM G0 subunits bearing carbonyl-linked nimesulide were obtained using *p*-nitrophenyl chloroformate as an efficient activator of both the hydroxyl group of the G3 core and nimesulide. The obtained conjugate is well soluble in water.

The G3 core is capable of binding ca 10 equivalents of G0 dendrimers to obtain a mixed generation megamer of molecular size within the range of other dendrimers, namely G3 (3.6 nm) < G3^gl^ (3.9 nm) < G4 (4.5 nm) **G3^G02N^** (5.1 nm) ≤ G4^gl^ (5.1 nm) < G5 (5.4 nm) < G5^gl^ (6.3 nm). The molecular size of such dendrimer renders them good candidates for crossing the cell membrane via endocytosis, and PAMAM dendrimer generation 3‒5 are used mostly as drug carriers. 

The megamer-nimesulide conjugate with **G3^gl^** core and *ca* 12 **G0^2N^** satellite dendrimers obtained was shown to effectively penetrate cells in micromolar concentrations. The conjugate has selective anticancer activity against human squamous carcinoma and glioma cells compared to normal human fibroblasts via inhibition of cell proliferation. Therefore, this megameric carrier provides a promising route for selective squamous carcinoma therapy. Additionally, megamers can be equipped with various drug and targeting molecules like biotin or folate, and other anticancer drugs that may additionally increase the effectiveness of anticancer therapy. 

## Appendix A

**Table A1:** Bond lengths and torsion angles in **1**.

Bond Lengths [Å]	Torsion Angles [°]
S1A O2A 1.424(2)	O2A S1A O1A 120.34(14)
S1A O1A 1.428(2)	O2A S1A N1A 103.73(14)
S1A N1A 1.691(3)	O1A S1A N1A 108.47(14)
S1A C1A 1.757(3)	O2A S1A C1A 109.51(16)
N1A C14A 1.379(4)	O1A S1A C1A 108.95(16)
N1A C2A 1.456(4)	N1A S1A C1A 104.64(15)
C2A C7A 1.364(4)	C14A N1A C2A 121.7(3)
C2A C3A 1.399(4)	C14A N1A S1A 119.7(2)
O3A C3A 1.373(4)	C2A N1A S1A 118.3(2)
O3A C8A 1.411(4)	C7A C2A C3A 121.0(3)
C3A C4A 1.377(4)	C7A C2A N1A 120.8(3)
O4A N5A 1.238(3)	C3A C2A N1A 118.2(3)
C4A C5A 1.377(4)	C3A O3A C8A 118.5(2)
O5A N5A 1.218(3)	O3A C3A C4A 124.3(3)
N5A C5A 1.483(4)	O3A C3A C2A 116.1(3)
C5A C6A 1.381(4)	C4A C3A C2A 119.6(3)
O6A C14A 1.359(4)	C5A C4A C3A 118.1(3)
O6A C15A 1.410(4)	O5A N5A O4A 124.1(3)
C6A C7A 1.392(4)	O5A N5A C5A 118.3(3)
O7A C14A 1.194(4)	O4A N5A C5A 117.6(3)
C8A C9A 1.365(5)	C4A C5A C6A 123.6(3)
C8A C13A 1.384(5)	C4A C5A N5A 116.8(3)
C9A C10A 1.391(5)	C6A C5A N5A 119.5(3)
C10A C11A 1.389(6)	C14A O6A C15A 117.2(2)
C11A C12A 1.372(6)	C5A C6A C7A 117.2(3)
C12A C13A 1.369(5)	C2A C7A C6A 120.5(3)
C15A C16A 1.373(4)	C9A C8A C13A 122.0(3)
C15A C20A 1.382(4)	C9A C8A O3A 121.5(3)
C16A C17A 1.397(4)	C13A C8A O3A 116.4(3)
C17A C18A 1.377(4)	C8A C9A C10A 119.0(4)
O18A N18A 1.233(3)	C11A C10A C9A 119.4(4)
N18A O19A 1.229(3)	C12A C11A C10A 120.3(4)
N18A C18A 1.459(4)	C13A C12A C11A 120.7(4)
C18A C19A 1.395(4)	C12A C13A C8A 118.6(4)
C19A C20A 1.390(4)	O7A C14A O6A 125.6(3)
S1 O2 1.424(2)	O7A C14A N1A 126.0(3)
S1 O1 1.427(2)	O6A C14A N1A 108.4(3)
S1 N1 1.687(3)	C16A C15A C20A 123.1(3)
S1 C1 1.748(4)	C16A C15A O6A 118.0(3)
N1 C14 1.393(4)	C20A C15A O6A 118.7(3)
N1 C2 1.448(4)	C15A C16A C17A 118.7(3)
C2 C7 1.378(4)	C18A C17A C16A 118.1(3)
C2 C3 1.394(4)	O19A N18A O18A 123.7(3)
O3 C3 1.368(4)	O19A N18A C18A 118.3(3)
O3 C8 1.412(4)	O18A N18A C18A 118.0(3)
C3 C4 1.384(4)	C17A C18A C19A 123.6(3)
C4 C5 1.380(5)	C17A C18A N18A 118.6(3)
O4 N5 1.231(4)	C19A C18A N18A 117.8(3)
O5 N5 1.224(3)	C20A C19A C18A 117.5(3)
N5 C5 1.480(4)	C15A C20A C19A 119.0(3)
C5 C6 1.385(4)	O2 S1 O1 119.91(14)
O6 C14 1.364(4)	O2 S1 N1 103.87(14)
O6 C15 1.409(4)	O1 S1 N1 108.49(13)
C6 C7 1.391(4)	O2 S1 C1 109.37(17)
O7 C14 1.189(4)	O1 S1 C1 108.88(16)
C8 C9 1.375(5)	N1 S1 C1 105.29(15)
C8 C13 1.380(5)	C14 N1 C2 122.4(3)
C9 C10 1.381(5)	C14 N1 S1 117.9(2)
C10 C11 1.379(5)	C2 N1 S1 118.8(2)
C11 C12 1.369(5)	C7 C2 C3 121.0(3)
C12 C13 1.375(5)	C7 C2 N1 120.3(3)
C15 C20 1.369(4)	C3 C2 N1 118.6(3)
C15 C16 1.387(4)	C3 O3 C8 117.8(2)
C16 C17 1.388(5)	O3 C3 C4 123.9(3)
C17 C18 1.385(4)	O3 C3 C2 116.0(3)
O18 N18 1.228(4)	C4 C3 C2 120.1(3)
N18 O19 1.233(3)	C5 C4 C3 117.3(3)
N18 C18 1.468(4)	O5 N5 O4 123.8(3)
C18 C19 1.381(4)	O5 N5 C5 118.5(3)
C19 C20 1.384(5)	O4 N5 C5 117.8(3)
Cl1 C100 1.737(4)	C4 C5 C6 124.2(3)
Cl2 C100 1.765(4)	C4 C5 N5 116.6(3)
Cl3 C100 1.754(4)	C6 C5 N5 119.1(3)
	C14 O6 C15 115.5(2)
	C5 C6 C7 117.2(3)
	C2 C7 C6 120.1(3)
	C9 C8 C13 121.8(3)
	C9 C8 O3 121.0(3)
	C13 C8 O3 117.2(3)
	C8 C9 C10 118.6(3)
	C11 C10 C9 120.2(4)
	C12 C11 C10 120.1(3)
	C11 C12 C13 120.8(3)
	C12 C13 C8 118.5(3)
	O7 C14 O6 126.0(3)
	O7 C14 N1 125.6(3)
	O6 C14 N1 108.4(3)
	C20 C15 C16 122.7(3)
	C20 C15 O6 119.5(3)
	C16 C15 O6 117.8(3)
	C15 C16 C17 118.5(3)
	C18 C17 C16 118.1(3)
	O18 N18 O19 123.8(3)
	O18 N18 C18 118.4(3)
	O19 N18 C18 117.8(3)
	C19 C18 C17 123.2(3)
	C19 C18 N18 119.0(3)
	C17 C18 N18 117.7(3)
	C18 C19 C20 117.9(3)
	C15 C20 C19 119.4(3)
	Cl1 C100 Cl3 111.1(2)
	Cl1 C100 Cl2 110.9(2)
	Cl3 C100 Cl2 110.6(2)

**Table A2:** Molecular weight parameters determined using GPC (see Figure A2 for description).

Species	M_n_	M_w_	M_z_	M_p_	M_w_/M_n_
**G3^G0^b**	11 860	18 980	30 930	14 370	1.60
**G3^G0^a**	11 010	15 050	20 220	14 920	1.37
**G3^G02N^**	13 740	22 400	37 230	17 310	1.63

**Table A3:** Molecular size of GM^gl^ and G3^G02N^ measured using DLS method.

Species	Diameter [nm]
G2^gl^	3.24 ± 0.11
G3^gl^	3.94 ± 0.11
G4^gl^	5.12 ± 0.12
G5^gl^	6.35 ± 0.14
G3^G0N^	5.07 ± 0.16
